# Examining the Use of Pill Swallowing in Dysphagia Assessment: A Survey of Common Practices

**DOI:** 10.1007/s00455-025-10846-y

**Published:** 2025-06-05

**Authors:** Meg Wood, Mary Gorham-Rowan, Ruth Renee Hannibal, Katherine Lamb, Michelle Cox

**Affiliations:** 1 Department of Communication Sciences and Disorders, Valdosta State University, Valdosta, USA; 2 Meadows Memorial Health Hospital, Vidalia, USA

**Keywords:** Dysphagia, Swallowing, Medication

## Abstract

Pill swallowing and medication administration can be problematic in patients with difficulty swallowing and can lead to poor medication compliance. The extent to which speech-language pathologists (SLPs) consider pill swallowing during videofluorographic assessments, however, is not known. A survey focusing on SLP practices, knowledge, and opinions about assessment of pill swallowing and subsequent medication administration decisions in patients with dysphagia was distributed to SLPs via American Speech-Language-Hearing Association (ASHA) special interest divisions and an online forum. Results from the survey revealed that while 53% of respondents agreed that pill swallowing should be included during dysphagia assessment, only 28% routinely do so. However, 58% of the SLPs reported making recommendations regarding medication administration based on the results of the videofluorographic exam. Further research should focus on best practices for pill swallowing assessment as well as exploring interventions for pill swallowing difficulty within the dysphagic population so that adverse events can be minimized, and patient outcome maximized.

## Introduction

Dysphagia is defined as difficulty swallowing [1]. Newman et al. [2] reports that 15–40% of the population demonstrates symptoms of dysphagia. This number is likely to be higher among older adults that suffer from diseases or chronic illnesses that contribute to dysphagia [3]. The presence of dysphagia often complicates health issues as well as quality of life [4]. As a result, the management and treatment of dysphagia can be complex and requires an accurate diagnosis. Not including the evaluation of pill swallowing in dysphagia assessment can contribute to complications associated with poor medication administration or patient noncompliance; however, very little information is available regarding pill swallowing evaluation standardization and treatment [5, 6].

An instrumental examination of swallowing is preferred as it allows for visualization of the swallow and structures involved in the process. An instrumental assessment may include videoendoscopy, videofluoroscopy (VFSS), and/or pharyngeal manometry [7, 8]; however, VFSS remains the “gold standard” of dysphagia assessment. Standardization with the VFSS is still lacking in the clinical setting [9]. Quantitative measures and published protocols are available but are time consuming in the clinical setting [10].

One area of concern that is minimally addressed in the literature is the evaluation of and use of regarding medication intake among patients with dysphagia [5, 11]. Although some patient-reported outcome measures include questions about pill swallowing, such as the EAT-10 [12] and the PILL-5 [13], pill swallowing is not a routine practice in standardized VFSS assessments [11], and the extent to which it’s included in clinician-developed protocols is unknown. Since evaluations dictate treatment, the inclusion of pill swallowing during VFSS should become standardized practice. During the administration of pill swallowing, the clinician can identify physiological impairments and then implement treatment options. Postural changes, compensatory techniques, and/or swallowing maneuvers may aid in the successful swallow of the pill [5, 11].

Oral intake is the predominant method for medication intake as it is less invasive and convenient [14]. Oral medication comes in different sizes and a variety of forms, including tablets, capsules, elixirs, sublingual tablets and films, and suspensions [15]. The solid oral dosage forms remain the most prevalent as they are cheaper and less complex to manufacture [16]. However, this form is the type that is the most difficult to swallow. Individuals without dysphagia report difficulty swallowing pills that are too large or too small [17] while oval tables are easiest to swallow [18]. These issues are likely exacerbated in individuals with dysphagia [16].

Patients with dysphagia exhibit longer swallow durations, more swallows, greater need for fluid to clear tablets, and an overall higher risk of aspiration or penetration during pill swallowing [16]. Difficulty in swallowing pills can lead to non-compliance [19]. Marquis et al. [20] found that nearly 23% of participants who had difficulty swallowing medication would compromise their adherence to their prescription medication and 63% did not inform their doctors or pharmacists of the problem. Non-compliance of medication is now recognized as a barrier to treatment and can contribute to disease progression and worsening of symptoms, which relate to increased healthcare costs, increased risk of aspirating medication, and decreased overall quality of life [16]. Medication intake should be a priority when evaluating dysphagia. Thus, this study aimed to gain perspective and knowledge regarding current practices in dysphagia assessment and management regarding medication intake. Although published protocols do not include pill swallowing as a feature, it was hypothesized that the majority of SLPs would evaluate pill swallowing during VFSS given that clinicians are trained to complete thorough evaluations to improve overall health and function. A second hypothesis was that SLPs would use this information to make recommendations regarding medication delivery.

## Methods

### Participants

Practicing SLPs were recruited through American Speech-Language-Hearing Association (ASHA) Special Interest Groups (SIGs), including Swallowing and Swallowing Disorders (SIG 13), Administration and Supervision (SIG 11), Gerontology (SIG 15), and Neurogenic Communication Disorders (SIG 2). Participants were also recruited from a dysphagia social media online forum group, *The Dysphagia Squad,* which has since been renamed *Dysphagia ProConnect.* The group consists of SLPs aiming to assist and provide resources for other SLPs that work with dysphagia patients. Inclusion criteria for all participants consisted of (a) certified SLPs (b) who have assessed and treated dysphagia patients within the last year, and (c) have or had access to use of VFSS for assessment.

### Survey Development

An online survey was developed via Qualtrics based on current SLP assessment practices and current literature regarding the use of pill swallowing in the evaluation process. The survey consisted of 30 questions, encompassing four sections, including participant demographics, VFSS protocols, evaluation of pill swallowing, and practices regarding recommendations. Questions regarding utilization of VFSS protocols and other standardized assessments were included as well but are beyond the scope of this study. These sections included a variety of response options including yes/no, multiple choice, and open-ended options. The survey was initially sent to 10 SLPs colleagues of the first author; the data and feedback obtained from these surveys were not included in the results but rather were used to refine the survey.

### Procedures

Approval from the Valdosta State University Institutional Review Board was obtained prior to dissemination of the survey. Consent from ASHA as well as the forum administrator was also obtained prior to initiation of data collection. A link to the survey was posted to the ASHA SIGs and the dysphagia forum group for a period of six weeks. All responses collected from the survey were anonymous.

### Data Analysis

Results are presented using descriptive statistics from the Qualtrics platform. For open-ended and *other* responses, the responses were examined for similarity according to content and grouped into sub-categories.

## Results

A total of 343 speech-language pathologists responded to the survey. Four of the 343 did not hold state licensure; therefore, those responses were excluded from the study’s data. In addition, three SLPs had not assessed and treated dysphagia within the past year, so those responses were not calculated. The final exclusion criterion was based on current access to VFSS and 85 SLPs did not; these data were not analyzed beyond the demographics. As a result, 251 SLP responses were included in the data analysis. However, not all participants responded to each question in the study as participants were allowed to skip questions. The total number of responses varied per question.

### Demographics

The largest group of participants had 21 + years of service followed by those with 6–10 years of service and 3–5 years of service Most of the participants worked in the hospital setting with skilled nursing facility (SNF) as the second most frequent place of employment (see Table 1). Additional employment settings not specified on the survey included subacute rehab, memory care, residential facility for individuals with disabilities, mobile FEES provider, and university Most clinicians felt comfortable performing VFSS, with the majority only performing VFSS five times or less per week (see Table 2).

**Table 1:** Demographic information of respondents

Extent of experience	n = 316*
21 + yrs	73
16–20 yrs	32
11–15 yrs	48
6–10 yrs	67
3–5 yrs	58
Less than 3 yrs	38

Employment setting	N = 317*
Hospital	141
Inpatient rehab	29
Outpatient rehab	29
Skilled nursing facility	73
Home health	11
Private practice	7
School	0
Other	27

*Not all participants responded to these questions

**Table 2:** Skill level and frequency of VFSS

Self-reported skill level	n = 314*
Expert	82
Competent	149
Needs assistance	83

Frequency of VFSS	n = 303*
Less than 5/week	241
6–10/week	46
More than 10/week	16

*Not all participants responded to these questions

### Evaluation of Pill Swallowing

In contrast to the first hypothesis, only 58 (28%) of the participants reported assessing pill swallowing on a routine basis. The majority of participants (n = 128, 62% reported that they *sometimes* assessed pill swallowing during VFSS. while 25 respondents (10%) responded that they did not assess pill swallowing during VFSS. Even though most SLPs assessed pill swallowing, 65% (n = 127) of the participants revealed it was not part of their protocol. In regard to the availability of assessing pill swallowing, 93% (n = 188) of the SLPs indicated that they did have access to pill swallowing assessment.

When pill swallowing was assessed during VFSS, most SLPs utilized a barium pill (see Table 3). Other responses included the use of barium filled capsules, gel caps, or medications crushed with barium; only one participant reported using the patient’s own medication. More than half of the participants (56%, n = 120) indicated that pill swallowing should be routine in VFSS practice, while 33% (n = 72) reported *maybe* and 11% (n = 24) indicated *no*. The predominant reasons for assessing pill swallowing were due to patient complaints of difficulty swallowing medication; additional reasons were related to VFSS protocol or nursing input (see Table 4).

**Table 3:** Materials used for pill swallowing assessment

	n = 194*
Barium pill	176
Patient’s medication	1
Other	17

*Not all participants responded to this question

**Table 4:** Reasons for pill swallowing assessment

	n = 410*
Patient complaint	165
Esophageal sweep/scan	67
Routine in practice	60
Nursing reported difficulty	100
Other	18

*Participants were able to choose multiple responses for this question

With regard to trying compensatory strategies to facilitate pill swallowing, only 63 participants (30%) reported that they regularly implemented strategies during assessment while 54% (n = 111) stated that they sometimes attempted them. Compensatory strategies were not included by 30 (16%) of the respondents. However, the majority of participants (83%) did report trying different substances to aid in pill swallowing. These substances included water, applesauce, pudding, thickened liquids, and pill swallowing lubricant or gel (see Table 5).

**Table 5:** Substances to facilitate pill swallowing

	n = 410*
Water	158
Applesauce	127
Pudding	117
Thickened liquids	89
Pill swallowing lubricant/gel	11

*Participants were able to choose multiple responses for this question

### Recommendations

**Fig. 1:**
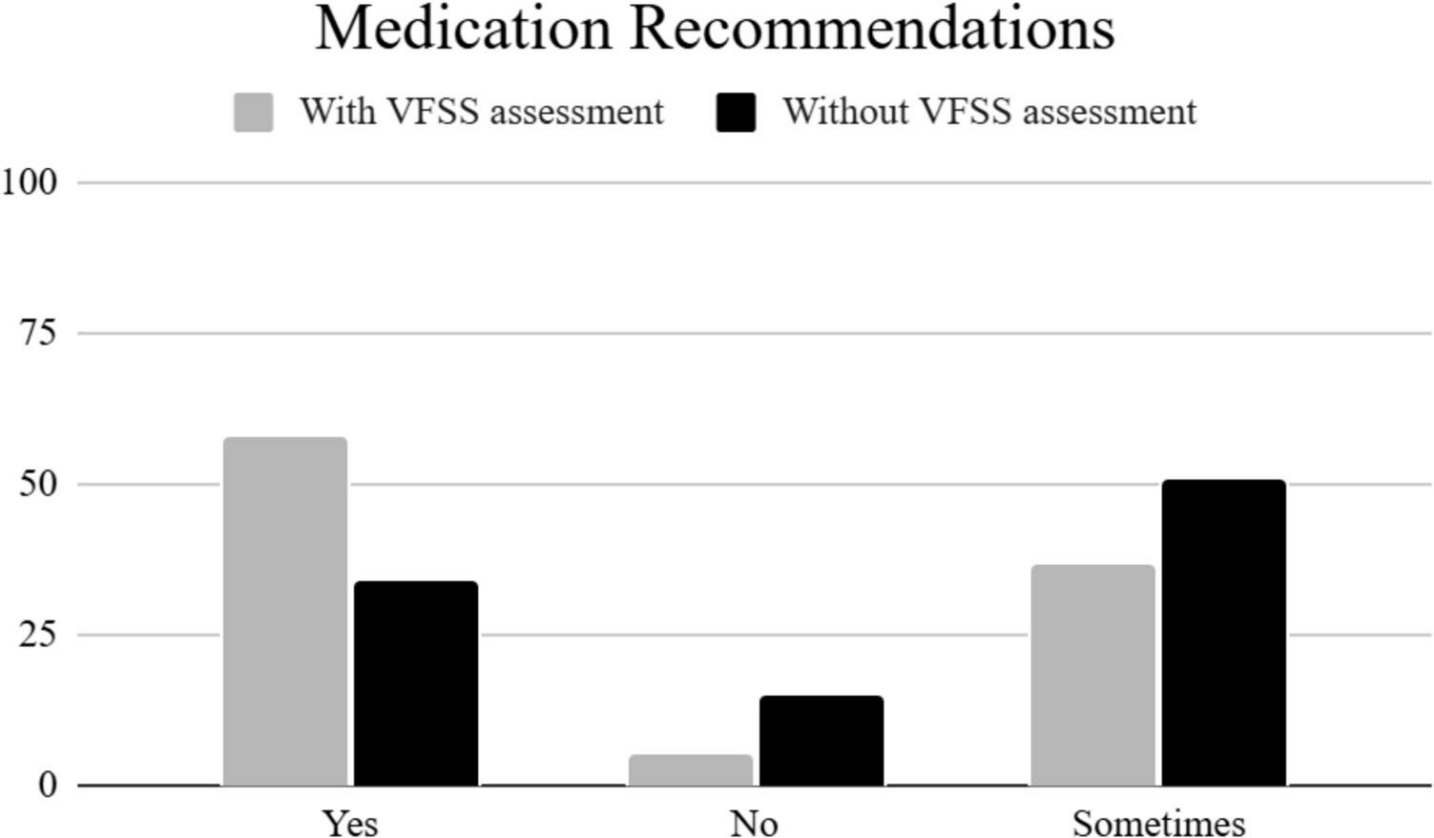
Percent of SLPs who reported making recommendations regarding medication administration based on inclusion of pill swallowing in VFSS

The final portion of the survey inquired about SLPs making recommendations based on assessment and pill swallowing ability. The majority of participants (92%, n = 205) reported they include pill swallowing assessment in their formal reports. More than half of the participants (58%, n = 111) made medication administration recommendations based on pill swallowing assessment during VFSS, which supports the second hypothesis of the study. Only 37% (n = 71) reported that they *sometimes* make recommendations, while 5% (n = 11) reported they don’t make recommendations based on pill assessment during swallowing. In contrast, a large number of participants reported that they also make recommendations without assessing pill swallowing, with 33% (n = 64) responding *yes* to this question and 52% (n = 103) responding *sometimes*. Only 15% (n = 30) of the respondents reported not making recommendations when pill swallowing was not assessed (see Fig. 1).

Participants that reported making medication administration recommendations were asked if they consulted with any other healthcare team members. Half of participants (50%, n = 128)) reported they consult nursing when making recommendations, while 33% (n = 84) consult the physician and 17% (n = 42) consult pharmacy.

Most SLPs (69%, n = 144) reported being able to write dietary changes for their facility, while 40% (n = 84) reported being able to write medication administration orders. The majority of participants (93%, n = 191) reported providing education to staff if medication administration recommendations were given.

## Discussion

The purpose of the current study was to obtain information concerning current clinical practices and opinions of SLPs regarding the inclusion of pill swallowing assessments during VFSS. The SLPs responding to this survey were experienced clinicians that felt competent in performing VFSS. The experience and level of self-reporting confidence from the participants lends to the fidelity of the responses given for this study.

### Evaluation of Pill Swallowing

Sixty-two percent of participants reported *sometimes* assessing, while 28% reported *regularly* assessing pill swallowing and 10% did not assess. While the majority did assess pill swallowing, this is an area lacking uniformity in dysphagia assessment. In fact, 64% indicate that pill swallowing is not listed as part of their established protocol that they follow and, as noted previously, it’s not part of standardized protocols [9]. As a result, most SLPs must determine the value of adding pill swallowing to their protocol. Again, these statistics imply the analytical skills required by SLPs to achieve maximal swallowing performance and evaluation with their patients.

When pill swallowing was assessed during VFSS, most (93%) use a barium pill as the medium and the same percentage report having access to assess pill swallowing. Most radiology suites have barium pills available as it is common procedure for the regular barium swallow; thus, it is an easy choice for SLPs to utilize. In addition to the accessibility of a barium pill, another advantage is the dissolvability of a barium pill. If the pill were to be aspirated or lodged in the pharynx, it would dissolve quickly, and barium is considered inert in the lungs.

In terms of opinions and feelings toward assessment of pill swallowing, only 53% reported that it should be routine in practice while 36% yielded *maybe.* The majority felt some level of interest and concern regarding the area of pill swallowing in dysphagia assessment, likely associated with the level of patient complaint related to pill swallowing. Indeed, patient complaint was the primary reason that SLPs would consider inclusion of pill swallowing during VFSS.

In addition to patient complaint, nursing reports of pill swallowing difficulty complaint, nursing reports of difficulty swallowing medication was frequently cited for assessing pill swallowing. As nurses administer medication to patients in most facilities, they will notice any difficulty with medication during the duration of hospitalization. Even if nursing doesn’t report difficulty with pill swallowing, SLPs should engage in this conversation with nursing to confirm that medications are being taken as prescribed as to ensure drug efficacy.

Inclusion of pill swallowing assessment during dysphagia assessment was also reported to be completed as part of an esophageal sweep or scan. This finding agrees with data reported by Watts et al. [9]. An esophageal sweep or scan is recommended during a VFSS to look for pill passage through the pharynx and esophagus. While this does relate to food and liquid passage through the gastrointestinal tract, esophageal sweeps do not usually yield recommendations for medication administration methods but rather are used for gaining a global perspective of the swallow sequence [9].

Finally, SLPs reported assessing pill swallowing in the evaluation process because it was routine in their practice. However, the reason for inclusion of pill assessment in these participants’ practice is unknown from the survey. It is surmised that SLPs routinely address pill swallowing because they understand the importance of being able to swallow medications safely.

Not only were the participants asked if they assessed pill swallowing and why, but also if they used any compensatory strategies in trying to help with pill swallowing function. As noted, compensatory strategies are tasks that can be implemented with the swallow to help aid in swallow safety. Only 27% of the participants reported regularly attempting compensatory strategies as in intervention with pill swallowing and 57% *sometimes* attempted them. The low percentage of regularly attempting compensatory strategies could be indicative of patients not needing an intervention to aid in pill swallowing and that the higher number of *sometimes* responses demonstrates the variability for the interventions as well.

Another intervention for trying to alleviate swallowing difficulty is diet modification. Diet modification and/or diet textures could be applied to improving pill swallowing as well. As a result, the participants were asked to list any different substances utilized in pill swallowing assessment. Most participants assessed pill swallowing with water, but others used applesauce, pudding, thickened liquids, and lubricants or gels, in order of decreasing frequency. There is a wide variety of mediums used to assess pill swallowing. While the survey did not ask participants if medications were placed whole in the substances or crushed, further investigation would be warranted as prior research has cautioned against crushing medications in substances due to alterations in pharmacokinetic properties [21, 22]. Perhaps an alternative means of assessing patients’ ability to swallow pills could be accomplished by utilizing different types and/or shapes of medications forms, e.g., oval vs round tablets, capsules, ODTs [22].

### Recommendations

The last portion of the survey focused on recommendations regarding medication administration. The responses from this section may be the most surprising results from the study. Only 58% of participants make recommendations for medication administration based on pill swallowing from the VFSS. An additional 37% will, at times, make recommendations. While this adds up to be the majority of participants, it’s interesting that many participants do not regularly make recommendations on medication administration from what is considered the gold standard of assessment. Although not addressed in this study, it would be worthwhile to examine if those participants don’t make recommendations because they don’t feel it is within their scope of practice or if they are not allowed to do so per their facility protocols.

Another interesting find from the study is that 34% of participants indicated that they make medication administration recommendations without assessing pill swallowing during VFSS. An additional 51% reported that they *sometimes* make recommendations without assessing pill swallowing. Again, this study didn’t ask for reasons as to why participants may do this, but several reasons could be offered. Access to pill swallowing evaluation is not likely a reason that pill swallowing is not assessed, as 93% of participants reporting they were able to do so. Perhaps a realistic explanation would be that the participants tend to use clinical judgment based on results from the other food and liquid trials that were completed during VFSS and infer how the patient may do with swallowing pills or medication. Given that physiological components of the swallowing process are observed during VFSS, participants could conclude that pill swallowing may or may not be difficult based on the function already visualized.

Related to making recommendations, the participants were asked if they consulted with other team members when making medication recommendations. Half of the participants indicated that they consulted with nursing. Only 32% indicated that they consult the doctor. Even fewer (17%) consult a pharmacist when making recommendations. It could be that, if pill swallowing was assessed and there was no difficulty, there is no reason to consult team members. The wording of this question may not have yielded the most accurate responses to changes in medication administration recommendations.

Moreover, these numbers may not be true indicators of interprofessional consultation. If the recommendations are for an outpatient VFSS, other team members wouldn’t necessarily be contacted if the patient lived at home. However, in extreme cases, even if the patient was living at home and could not easily swallow medications, some form of communication would at least be discussed with the primary care doctor.

A follow up question related to making recommendations for medication administration focused on dietary changes and medication orders. Most participants reported being able to write dietary changes, and nearly half reported being able to write medication administration orders. The fact that almost half of the participants can write medication administration orders implies the need that pill swallowing should be addressed during assessment. Finally, nearly all the participants reported that they do provide staff education if medication administration changes were recommended.

While this is the first study known to the author to survey SLPs’ practices toward VFSS protocols and pill swallowing assessment, there are several limitations to the study. Even though survey methodology is helpful for providing a general standing of practices currently in the field, this survey did not attempt to gain the reasoning for the actions listed by the participants. This study is simply a starting point for common trends in the field. Secondly, this study only focused on one form of dysphagia assessment, the VFSS. SLPs may be assessing pill swallowing during clinical assessments or videoendoscopy [21] if they do not have access to VFSS and the results could vary greatly. Finally, the SLPs were able to skip questions on the survey, resulting in several respondents not answering all questions, which may have biased the outcome of the study.

In conclusion, this study reported on the current practices and attitudes of SLPs toward pill swallowing assessment in dysphagia assessment, specifically VFSS. The study revealed marked variability in assessing pill swallowing during VFSS. However, the study also yielded that many SLPs understand the need for assessing pill swallowing by including it in their assessments despite a lack of standardization in the field.

As adherence to medication prescriptions are correlated with increased health outcomes, this area should continue to be studied in the dysphagic population as limited options are available [14]. In order to improve the compliance and effectiveness of medication administration in patients with dysphagia, a multidisciplinary approach should be considered that includes the patient, SLP, physician, nurse, and pharmacist. Open communication from patients regarding their ability to swallow and adhere to the medication regimen is needed. In addition, consultations among doctors, nurses, and pharmacists are suggested to ensure that dosages, forms, and frequencies best meet the needs of each patient. Finally, current practices regarding alternative administration of medication, such as crushing the medications and/or using thickened substances to facilitate swallowing, may not be the most effective means by which to deliver the medicine. Further research is needed regarding the use of compensatory strategies and swallowing maneuvers to aid in pill clearance.

## Data Availability

All data pertaining to this study have been summarized in the paper. The raw data are available upon request from the corresponding author.
